# Optimization of Vitamin Suppletion After Roux-En-Y Gastric Bypass Surgery Can Lower Postoperative Deficiencies: A Randomized Controlled Trial

**DOI:** 10.1097/MD.0000000000000169

**Published:** 2014-11-28

**Authors:** Kemal Dogan, Edo O. Aarts, Parweez Koehestanie, Bark Betzel, Nadine Ploeger, Hans de Boer, Theo J. Aufenacker, Kees J.H.M. van Laarhoven, Ignace M.C. Janssen, Frits J. Berends

**Affiliations:** From the Department of Surgery (KD, EOA, PK, BB, NP, TJA, IMCJ, FJB); Department of Internal Medicine, Rijnstate Hospital, Arnhem (HDB); and Department of Surgery, Radboud University Medical Centre, Nijmegen, The Netherlands (KJHMVL).

## Abstract

Iron, vitamin B12, and folic acid deficiencies are among the most common deficiencies occurring after laparoscopic Roux-en-Y gastric bypass (LRYGB). The present study evaluates the effectiveness of a specially designed multivitamin supplement (WLS Forte, FitForMe, Rotterdam, the Netherlands) specifically developed for LRYGB patients.

A triple-blind, randomized, 12-month study was conducted comparing WLS forte with a standard multivitamin supplement (sMVS) containing approximately 100% of the recommended daily allowance (RDA) for iron, vitamin B12, and folic acid. WLS Forte contains vitamin B12 14000% RDA, iron 500% RDA, and folic acid 300% RDA.

In total, 148 patients (74 in each group) underwent a LRYGB procedure. Baseline characteristics were similar for both groups. Per protocol analysis demonstrated that sMVS treatment was associated with a decline in ferritin (−24.4 ± 70.1 μg/L) and vitamin B12 (−45.9 ± 150.3 pmol/L) over 12 months, whereas in WLS Forte patients, ferritin remained stable (+3.2 ± 93.2 μg/L) and vitamin B12 increased significantly (+55.1 ± 144.2 pmol/L). The number of patients developing ferritin or vitamin B12 deficiency was significantly lower with WLS Forte compared with sMVS (*P* < 0.05). Iron deficiency (ID) was reduced by 88% after WLS Forte compared with sMVS. Adverse events related to supplement use did not occur.

An optimized multivitamin supplement is safe and reduces the development of iron and vitamin B12 deficiencies after LRYGB.

## INTRODUCTION

Bariatric surgery has proven itself as an effective treatment to establish sustained weight loss in morbidly obese patients, and to reduce obesity-related comorbidities such as type 2 diabetes mellitus, hypertension, and sleep apnea syndrome.^1–6^ The Roux-en-Y gastric bypass (RYGB) is one of the most frequently performed surgical procedures worldwide to induce weight loss.^7^ However, as a result of the created restriction of intake and partial bypass of the upper intestinal tract, it also increases the risk of nutritional deficiencies. Most commonly diagnosed postsurgical deficiencies are iron (47%–66%), vitamin B12 (37%–50%), folic acid (15%–38%), vitamin D (20%–51%), and calcium (± 10%).^8–25^ To prevent these postsurgical deficiencies, daily use of a multivitamin and mineral supplements are generally recommended^26^; however, guidelines vary and RYGB-specific, multivitamin supplements (MVS) are currently not available.^12,15,23,27^

Based on the literature and pilot studies performed in our hospital, a customized MVS for RYGB patients was developed (WLS Forte, FitForMe, Rotterdam, the Netherlands). The present study evaluates the effectiveness and safety of WLS Forte compared with standard MVS (sMVS, commercially available tablets) after RYGB in a triple-blind randomized controlled trial.

## PATIENTS AND METHODS

The study protocol was approved by the National Medical Ethics Review Committee of the Radboud University Medical Centre and Local Ethical Committee of the Rijnstate Hospital Arnhem (RHA), and was conducted in concordance with the principles of the Declaration of Helsinki. The study protocol was registered at the clinical trials registry of the National Institutes of Health (ClinicalTrials.gov; identifier NCT 01609387).

### Study Design

The present study was a triple-blind, randomized, clinical trial. The patients were randomized in 2 groups, receiving 2 different multivitamin supplements (standard multivitamin supplement and WLS Forte) after a primary laparoscopic Roux-en-Y gastric bypass (LRYGB) operation. Patients were included by bariatric surgeons, (supervised) specialized obesity nurses, or researchers at the outpatient surgery department of the RHA. RHA is a teaching hospital for surgical residents and a center of excellence in bariatric surgery.

### Patients

Patients who were scheduled for a LRYGB operation between June 2011 and March 2012 were invited for participation in the study and written informed consent was obtained from each patient.

Adult patients (age >18 years) with morbid obesity (body mass index [BMI] of >40 or >35 kg/m^2^with an obesity-related comorbidity) who met the criteria for bariatric surgery according to the National Institutes of Health Consensus Development Conference Panel for bariatric surgery^28^ were eligible for the study. Exclusion criteria were a secondary LRYGB operation or other type of a bariatric procedure.

### Surgical Procedure

All procedures were performed by 1 of 3 experienced bariatric surgeons (>750 procedures each). They performed an antecolic antegastric LRYGB, with a proximal gastric pouch of 30 mL, a biliopancreatic limb of 50 cm, and a Roux limb of 150 cm. All patients received low-molecular heparin (nadroparin 5700 IU daily) for 6 weeks and proton-pump inhibitor (omeprazol 40 mg daily) for 6 months, as part of our standard postoperative protocol.

### Intervention and Control

WLS Forte, a customized multivitamin supplement for RYGB patients, contains high doses of multiple vitamins and minerals, in particular iron (5 times recommended daily allowance [RDA]), folic acid (3 times RDA), and vitamin B12 (140 times RDA). A standard multivitamin supplement (sMVS, FitForMe) served as control and contains the compounds of interest in a dose equivalent to the RDA. The composition of both supplements is shown in Table 1. Both supplements were dosed as 1 capsule daily. In both groups, all patients also received calcium carbonate/cholecalciferol 500/400 tablets 3 times daily (a total of 1500 mg calcium and 1200 IU vitamin D).

**TABLE 1:**
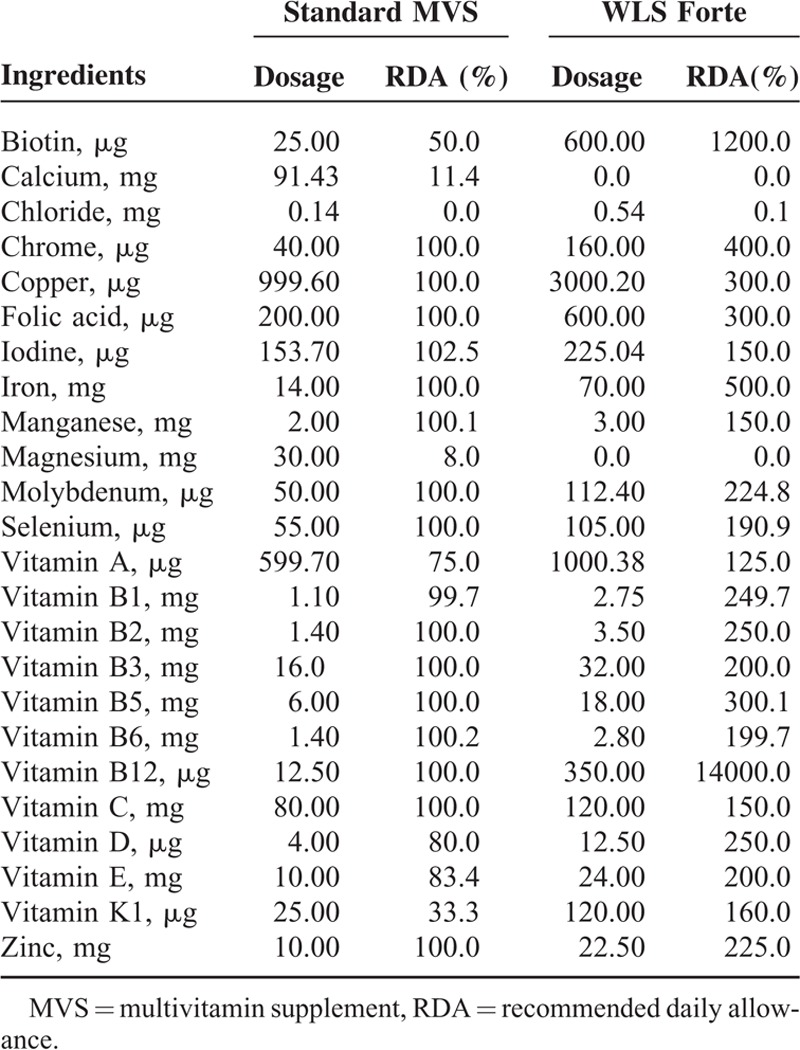
Dosages of Supplement Ingredients

### Randomization and Blinding

The allocation sequence was computer generated. Variable block schedule was used for randomization and conducted by the independent trial pharmacist. Allocation concealment was achieved by generating a randomization list that was only available to the pharmacist. All supplements were packaged in a nonmarked sealed box at the hospital pharmacy, which was similar for both supplements, and numbered according to the randomization list. This was performed before the inclusion of the participants. The included participants received the sealed box with the supplements according to the list, which was delivered by the pharmacist. Both supplements were similar in color, size, and taste. Therefore, patients, surgeons, and researchers were unaware of the type of the supplements. After the last visit of the last study patient, the randomization list was available for the research team. No earlier unblinding occurred. Patients with a nutritional deficiency before or during the study were treated with a predefined medication, independent of the trial group.

### Data Collection, Follow-Up, and Outcome

All patients followed a strict postoperative schedule consisting of 17 visits in the first year, and on each visit patients were encouraged to keep taking their supplements. Standard laboratory blood tests were performed at baseline, and at 6 and 12 months. This included a complete blood count, calcium, phosphate, magnesium, zinc, albumin, iron, total iron-binding capacity, ferritin, folic acid, vitamin B12, 25-hydroxyvitamin D (25-OHD), parathyroid hormone (PTH), vitamin B1, and vitamin B6 (normal ranges are presented in tables).

Primary outcome variables were the percentage of iron, folic acid, and vitamin B12 deficiencies developed during the 12 months after LRYGB. Iron deficiency (ID) was defined as a serum ferritin <20 μg/L, folic acid deficiency if the level was < 9.0 nmol/L, and vitamin B12 deficiency if the level was < 150 pmol/L. Anemia was defined as hemoglobin level of <7.4 mmol/L for females and <8.4 for males. Vitamin D deficiency was diagnosed if 25-OHD was <50 nmol/L, hypocalcaemia if serum total calcium was <2.1 mmol/L, and zinc deficiency of the serum level was <9.2 μmol/L. Calcium data are shown as calcium levels corrected for albumin (Ca_corr_), according to the following equation: Ca_corr_ = Total Calcium – (0.025 x albumin) + 1.

### Correction of Deficiencies

Preoperative deficiencies for iron, folic acid, vitamin B12, and vitamin D were treated with predefined medication until 2 months preoperatively so that it would not intervene with the postoperative multivitamin supplements. If a postoperative deficiency occurred, it was recorded for the purpose of this study. Subsequently the deficiency was corrected.

ID with or without anemia was treated with ferrous gluconate 695 mg 3 times daily for 3 months. Vitamin B12 deficiency was corrected with intramuscular injection of 1000 μg hydroxocobalamin once every 2 months for 12 months. Vitamin D deficiency was corrected with oral solubilized cholecalciferol^FNA^ 50,000 IU/mL. First, a loading dose (IU) was calculated with the formula: 40 × (75 – actual serum 25-OHD level) × body weight in kilograms, as described previously.^29,30^ Thereafter, a maintenance dosage of 25,000 IU cholecalciferol^FNA^ per week was prescribed for a period of 3 months. For other deficiencies the endocrinologist was consulted.

### Analysis and Sample Size Calculation

Intention-to-treat analysis was conducted to present the outcomes. Additionally, per protocol analysis was performed, excluding patients who received additional medication because of a nutritional deficiency during the study.

All data were analyzed using IBM SPSS Statistics (IBM Software Group, Chicago, IL, USA) 20 for Windows. Data are expressed as mean (±standard deviation), unless otherwise specified. Differences between groups were calculated using Student *t*-test for continuous data and chi-square test for ordinal/nominal data. A *P* value <0.05 was considered statistically significant.

Sample size calculation was performed by the epidemiologist of the Research Department of RHA using Openepi.com. Sample size calculation was based on the number of patients developing ID. To detect a 25% reduction of ID 12 months after surgery, with 95% sensitivity and a power of 90%, a minimum of 56 patients per group were needed. Taking into account a 10% dropout and 15% of cases excluded because of ID diagnosed and treated at 6 months, it was decided to include 75 patients per treatment group.

## RESULTS

Two patients were excluded after randomization: 1 patient in the WLS Forte group because he underwent a sleeve gastrectomy instead of a LRYGB because of multiple adhesions during surgery, and 1 patient in the sMVS group because he cancelled the scheduled operation procedure. In total, 148 patients (74 in each group) underwent a LRYGB and were included for analysis. Baseline patient characteristics are shown in Table 2. Both groups were similar with respect to age, sex, weight, BMI, and preoperative deficiencies. However, dyslipidemia was twice as frequent in the WLS Forte group as compared with the sMVS group (*P* = 0.04).

**TABLE 2:**
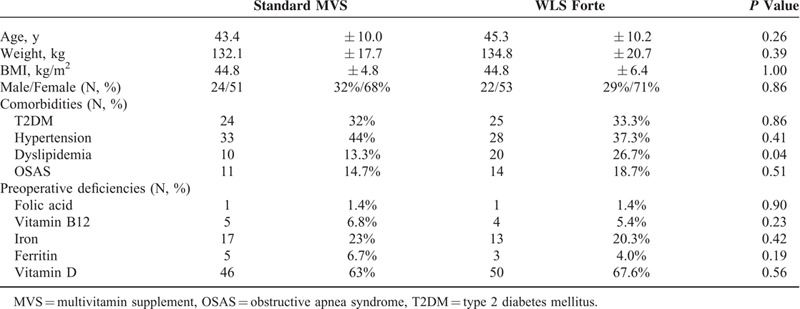
Baseline Characteristics of Patients

Weight Loss

The degree of weight loss over 12 months was similar in both groups. Weight dropped to 90.6 ± 17.4 kg in the sMVS group versus 93.8 ± 16.9 kg in the WLS Forte group (*P* = 0.24), respectively. Percentage excess weight loss, defined as weight loss divided by excess weight based on ideal body weight at BMI 25 kg/m^2^, were after 12 months 72.5 ± 20.9 kg/m^2^ versus 72.1 ± 23.2 kg/m^2^ for MVS and WLS Forte (*P* = 0.92), respectively.

### Iron, Vitamin B12, and Folic Acid Deficiency

In Table 3, laboratory serum levels of hemoglobin metabolism are shown. Mean hemoglobin levels at baseline were 8.5 ± 0.7 mmol/L (sMVS) and 8.6 ± 0.7 mmol/L (WLS Forte) (*P* = 0.24), and did not change over 12 months. Mean cell volumes also did not change (data not shown). In total, 10 (6.8%) patients had anemia preoperatively: 6 (8.1%) patients in the sMVS and 4 (5.4%) in the WLS Forte group (*P* = 0.50). Three of them (1.4%), 2 patients in sMVS and 1 patient in WLS Forte, had ID anemia. After 12 months postoperatively, 3 patients (4.3%) versus 5 patients (7.4%) had anemia (*P* = 0.75), respectively. Of these patients, 2 patients had anemia de novo in both groups. Two patients (1.5%), 1 patient in each group, had ID anemia.

**TABLE 3:**
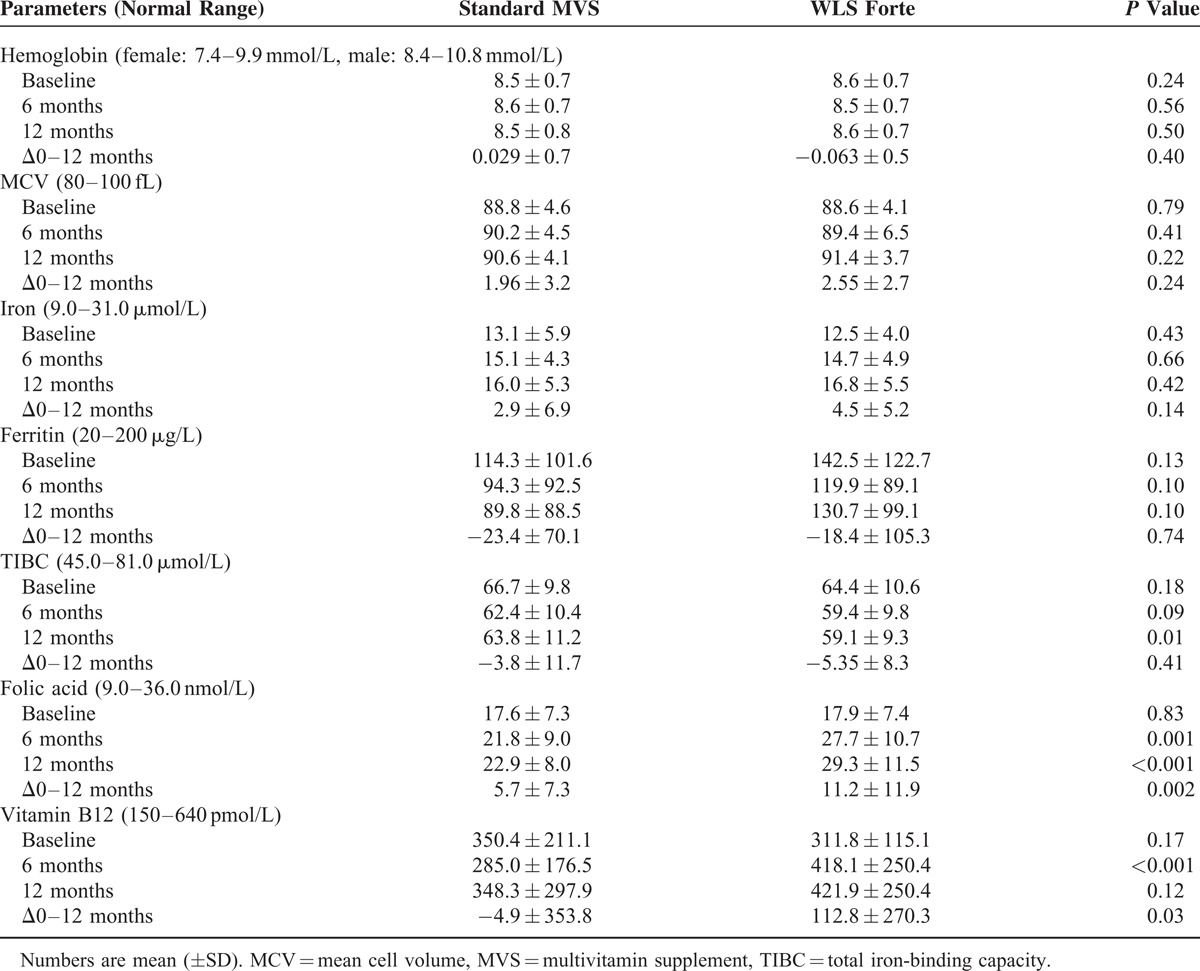
Evaluation of Hemoglobin Metabolism

The total number of patients developing ferritin deficiency during follow-up was 8 (10.7%) in sMVS and 1 (1.3%) in WLS Forte (*P* = 0.03). In total, 55 (37.2%) patients, 28 (37.8%) in the sMVS group and 27 (36.5%) in the WLS Forte group, received additional iron medication at any time during the 12 month follow-up. Results after exclusion of these patients are shown in Table 4. Mean serum ferritin decreased by 18.4 ± 61.8 μg/L in the sMVS group, but remained stable in the WLS Forte group (*P* = 0.08).

**TABLE 4:**
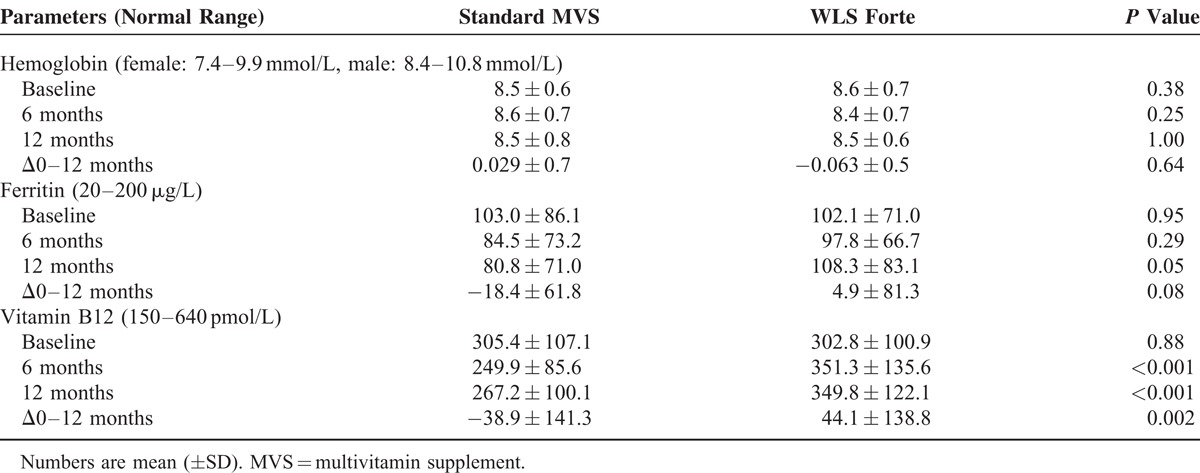
Results of Hemoglobin Metabolism, After Exclusion of Patients Who Received Additional Iron and/or Vitamin B12 Medication

At baseline, vitamin B12 deficiency was diagnosed in 9 (6.1%) patients, that is, 5 (6.8%) patients in the sMVS group and 4 (5.4%) patients in the WLS Forte group. These patients received vitamin B12 injections by protocol. In total, 27 (18.2%) additional patients were treated with vitamin B12 injections at any time during the 12-month follow-up: 17 (23%) in the sMVS group and 10 (13.5%) in the WLS Forte group (*P* = 0.14). The results obtained after exclusion of these patients receiving vitamin B12 injections are shown in Table 4. Mean vitamin B12 serum levels decreased by 38.9 ± 141.3 pmol/L in the sMVS group and increased by 44.1 ± 138.8 pmol/L in the WLS Forte group (*P*<0.001) after 12 months, and as a result mean vitamin B12 blood serum levels at 6 months and 12 months were significantly higher with WLS Forte compared with sMVS (*P* < 0.05). After 12 months, vitamin B12 deficiency had developed in 5 (7.9%) patients receiving sMVS versus 1 patient (1.6%) in the WLS Forte group (*P* = 0.207).

Mean serum folic acid levels were similar at baseline: 17.6 ± 7.3 nmol/L and 17.9 ± 7.4 nmol/L (*P* = 0.83) for sMVS and WLS Forte, respectively. In both groups, 1 patient (1.4%) had folic acid deficiency at baseline. During the study period, folic acid deficiency developed in 5 (6.8%) patients taking sMVS and 2 (2.7%) patients in the WLS Forte group (*P* = 0.441). After 6 and 12 months, mean serum folic acid levels were significant higher in the WLS Forte group (*P* < 0.05).

### Vitamin D Metabolism

In total, 96 (65.3%) patients, 46 (63.0%) patients in the sMVS group and 50 (67.6%) patients in the WLS Forte group (*P* = 0.56), had vitamin D deficiency before surgery. Mean serum vitamin D levels were 42.5 ± 17.9 nmol/L and 44.3 ± 20.6 nmol/L at baseline (*P* = 0.58), respectively. Vitamin D deficiencies were corrected according to the protocol and this resulted in mean 25-OHD levels close to the target of 75 nmol/L in both groups. The mean loading dose was 226,087 ± 60,442 IU with a maintenance dose of 25,000 IU/month. At 12 months, 7 (10.1%) patients in the sMVS and 12 (18.5%) patients in the WLS Forte group had vitamin D deficiency (*P* = 0.168). Excluding the patients who received extra vitamin D supplementation additional to the standard suppletion, mean vitamin D levels at baseline were 61.8 ± 10.8 versus 66.8 ± 19.3 nmol/L (*P* = 0.29) for sMVS and WLS Forte; after 12 months 84.6 ± 20.8 nmol/L versus 83.4 ± 24.8 nmol/L (*P* = 0.87), respectively. There were no differences in PTH levels and calcium levels between the 2 groups (*P* ≥ 0.05), although PTH levels rose by 1.26 ± 2.39 and 1.75 ± 3.1 pmol/L. Table 5 shows serum blood tests of the vitamin D metabolism at fixed time points.

**TABLE 5:**
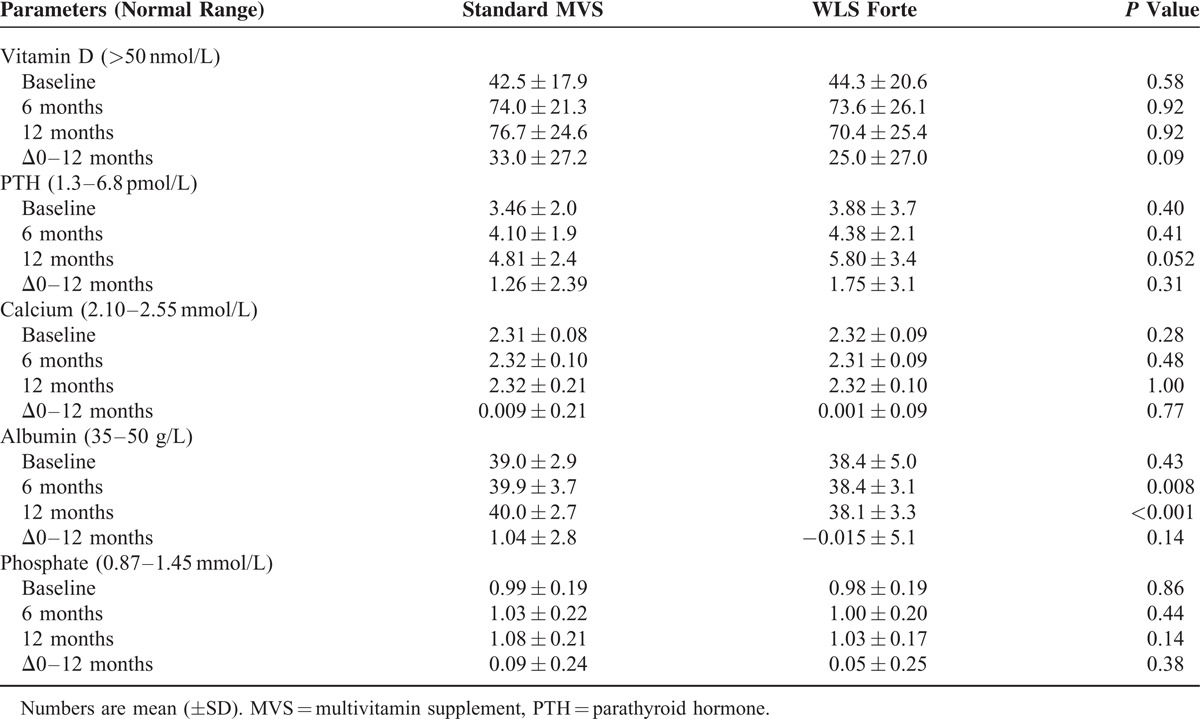
Results of Calcium and Vitamin D Metabolism

### Other Vitamins and Minerals

Results of monitoring vitamin B1, vitamin B6, zinc, and magnesium are shown in Table 6. Vitamin B6 levels were at baseline 68.6 ± 22.1 nmol/L versus 74.9 ± 22.8 nmol/L for the sMVS and WLS Forte groups (*P* = 0.13), respectively. There were no vitamin B6 deficiencies observed at baseline. In total, 15 (10.1%) patients, 5 (6.8%) patients in sMVS versus 10 (13.5%) patients in WLS Forte (*P* = 0.173), had elevated serum vitamin B6 levels at baseline. After 12 months, there were no deficiencies in either group and elevated serum vitamin B6 levels were observed in a total of 52 (40.6%) patients, that is, in 21 (31.8%) patients in the sMVS group and 31 (50%) patients in the WLS Forte group (*P* = 0.036). In 1 patient, an extremely high level of vitamin B6 (2777 nmol/L), without any neurological symptoms, was detected in the WLS Forte group; however, this patient had used additional multivitamin supplements on advice of his general practitioner in addition to the study medication. Two months after withdrawal of these additional medications, the serum level had dropped to 110 nmol/L.

**TABLE 6:**
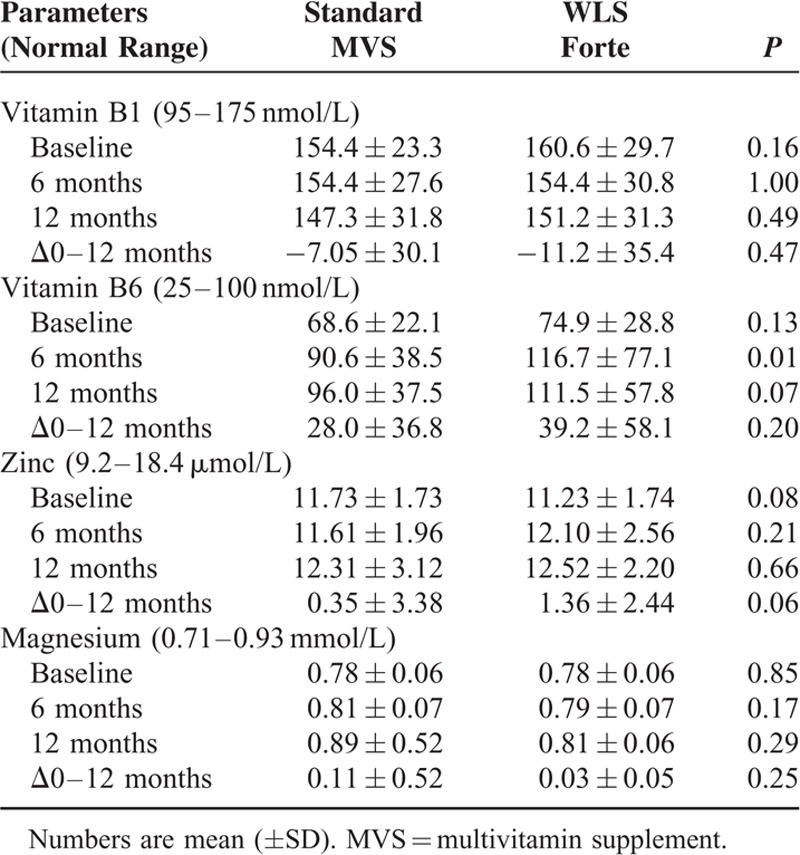
Results of Monitoring Vitamin B1 and B6, and Minerals Zinc and Magnesium

For vitamin B1, zinc, phosphate, and magnesium, no differences were observed between the groups.

## DISCUSSION

The present study illustrates that a multivitamin supplement specifically developed to prevent deficiencies in LRYGB patients has advantages over standard supplementation. sMVS was associated with a substantial decline in serum ferritin and vitamin B12 levels over 1 year, whereas, in patients on WLS Forte, ferritin remained stable and vitamin B12 increased significantly. Moreover, the numbers of patients developing ferritin or vitamin B12 deficiencies were significantly fewer with the LRYGB-specific multivitamin. These results were achieved by raising the iron content from 1 to 5 times RDA and vitamin B12 from 1 to 140 times RDA. No benefit was found with respect to folic acid, despite a difference between the supplements in folic acid content of 1 and 3 times RDA.

### Iron Deficiency

Decreased acid production in the small gastric pouch, exclusion of the duodenum and proximal part of the jejunum, use of proton-pump inhibitors during the first months of surgery, and intolerance of iron-rich foods, such as red meat, are the main factors increasing the risk of iron deficiency.^27,31^ ID in the first year after LRYGB has been reported to occur in 44% to 66% of patients not using supplements. These figures should be interpreted with caution because of marked differences in the definition of iron deficiency between these studies. Sometimes serum iron levels are erroneously used to diagnose iron deficiency. Serum iron levels do not reflect the body iron store. In the absence of inflammation, serum ferritin is the gold standard. Standardized supplementation of 14 mg iron daily reduced the incidence of iron deficiency in the first year after LRYGB to 11%. The American Society of Metabolic and Bariatric Surgery (ASMBS) guidelines advise a minimum of 18 mg iron per day for patients after RYGB. An additional minimum of 18 to 27 mg per day elemental iron is advised for high-risk patients including menstruating women.^32^ However, Vargas-Ruiz et al^22^ demonstrated that 18 mg iron per day was insufficient in LRYGB patients. ID was diagnosed in 20% of the patients after 12 months and rose to 55% after 3 years. This is in line with the observation of Aarts et al^8^ where ID was diagnosed in 21% of the patients despite a daily intake of a minimum of 21 mg iron. In addition, female patients had a higher risk of developing ID than men (38% versus 17%, *P* = 0.02). Brolin et al^11^ demonstrated, in a double-blind, randomized controlled trial, the effectiveness of 320 mg ferrous sulphate twice daily (total of 130 mg elemental iron) in young women who underwent a RYGB for prevention of ID.

Based on the literature that advises 40 to 65 mg intake of iron in men and 100 mg in female patients after RYGB, the iron content in WLS Forte was increased to 70 mg, that is, 5 times RDA.^8,23,33,34^ In the present study with a male to female ratio of 1 : 2.3, this was sufficient to maintain serum ferritin at baseline level, whereas, in patients on sMVS, ferritin steadily decreased over the year. The fact that the difference in ferritin levels at 12 months did not reach statistical significance is attributed to a lack of power in this study.

### Vitamin B12 Deficiency

Vitamin B12 in food is bound to proteins, and needs to be liberated by the action of hydrochloric acid, pepsin, and pancreatic enzymes. This process is compromised after RYGB. In addition, intrinsic factor release is also reduced, which further compromises vitamin B12 absorption. Finally, preoperative vitamin B12 deficiencies are not uncommon in the morbidly obese, probably because of their dietary habits. In the present study, 9% of patients had vitamin B12 deficiency preoperatively. Published data of postoperative vitamin B12 deficiencies diagnosed after variable periods of time vary from 10% to 50%.^11,21,22,24^ Vargas-Ruiz et al^22^ reported a post-RYGB incidence of vitamin B12 deficiency of 10% after 12 months and 18% after 3 years, despite the use of a multivitamin containing cobalamin 6 μg daily. Brolin et al^11^ reported that 37% of the patients had vitamin B12 deficiency after a mean follow-up period of 42 months. ASMBS guidelines advise vitamin B12 supplements of 350 to 500 μg daily, and as-needed addition of intramuscular injections of 1000* *μg per month.^32^ WLS Forte contains high doses of 350 μg vitamin B12, which is 14,000% ADH. After 12 months, vitamin B12 hypervitaminosis was observed in 9 patients on WLS Forte compared with 7 patients in sMVS (*P* = 0.58). No adverse events of vitamin B12 hypervitaminosis were observed during the study period.

ID, vitamin B12 deficiency, and folic acid deficiencies are associated with anemia after LRYGB.^8,11,22^ A previous study of our group demonstrated high incidence of deficiencies for iron (66%), vitamin B12 (50%), and folic acid (15%) in patients with anemia after LRYGB.^8^ Based on these results, minimal daily intake of 65 mg of iron in males and 100 mg in female patients, 350 μg of vitamin B12, and 400 μg of folic acid were advised after LRYGB. In our current study, the prevalence of anemia was, in total, 11.7% after 12 months, that is, in 4.3% on sMVS versus 7.4% on WLS Forte (*P* = 0.75). However, only 1 patient had an ID anemia in the sMVS group. The fact that 23% of the patients received additional oral iron medication at baseline and 10% received vitamin B12 injections at baseline or 6 months may have resulted in lower nutrient-related anemia. A previous study of Brolin et al^35^ showed also reduction in ID with additional iron supplements; however, incidence of anemia was not reduced compared with placebo. Other pathophysiological aspects play probably a role in anemia after RYGB surgery.

### Vitamin D

Vitamin D, a fat-soluble vitamin, can be produced in the skin under influence of sunlight, and ingested through the diet (eg, certain fish and milk products) or by dietary supplements.^36,37^ It is metabolized in the liver into 25-OH-D, the best parameter for patients’ vitamin D status.^10,33^ Several studies claimed high incidence of vitamin D (34%–73%) deficiency after RYGB^38–40^; however, the exact mechanisms of postoperative vitamin D deficiency are not completely clear yet. Obesity itself seems to be a risk factor of developing vitamin deficiency.

Despite the high incidence of postoperative vitamin D deficiency, optimal prevention strategies are lacking. The ASMBS guidelines advise daily intake of 2000 IU vitamin D. In addition, 1500 to 2000 mg per day of calcium was recommended.^32^ In contrast to these guidelines, reports in the literature advise postoperative vitamin D supplements varying between 320 IU and 2000 IU per day,^12,27,41,42^ and a calcium intake of 1000 to 1500 mg daily.^27,34,42^

Carlin et al^43^ demonstrated that despite daily intake of 800 IU vitamin D, 44% of the population still remained insufficient for vitamin D. Goldner et al^41^ conducted a prospective, randomized controlled trial comparing 3 different doses of vitamin D supplementation of 800 IU, 2000 IU and 5000 IU daily following RYGB. The postoperative increase of mean serum vitamin D level at 12 months was higher with higher doses (800–5000 IU) of vitamin D supplement. Therefore, a minimum of 2000 IU daily intake of vitamin D was advised. However, Signori et al^44^ reported that daily use of prophylactic vitamin D of 1200 to 2000 IU did not prevent postoperative vitamin D deficiency.

In the current study, all patients received 1500 mg calcium carbonate and 1200 IU vitamin D daily as standard postoperative protocol. Furthermore, sMVS (control group) contained 160 IU (4 μg) vitamin D, whereas WLS Forte contained 500 IU (12.5 μg). In total, 29% of the cohort had developed vitamin D deficiency after 12 months, without significant differences between the 2 supplementation regimes. At 12 months, 10% in the sMVS group and 19% of patients in the WLS Forte group had developed vitamin D deficiency (*P* = 0.168).

A previous study from our group demonstrated a 25% reduction of peak cholecalciferol levels after intake of oral cholecalciferol of 50,000 IU, corresponding with impaired absorption.^30^ These results in combination with previously reported studies suggest that vitamin D deficiency after LRYGB is difficult to prevent with a standard multivitamin supplement, and that much higher doses of vitamin D are needed to prevent vitamin D deficiency.

### Other Vitamin and Minerals

In our study, no differences were found in vitamin B1, vitamin B6, zinc, magnesium, or phosphate serum levels between standard MVS and WLS Forte. A notable finding is the high incidence (10%) of hypervitaminosis of vitamin B6 before the surgery. In addition, in total, 41% had high levels of vitamin B6 after surgery, which was significantly higher for WLS Forte compared with sMVS at 12 months. Increased levels of vitamin B6 were also observed in the literature,^45–47^ but no adverse events were reported. As 100% ADH of vitamin B6 is associated with increased serum levels after surgery and no deficiencies, no higher concentrations were needed as in WLS Forte. Therefore, vitamin B6 dose was decreased to 0.98 mg (70%) RDA in the WLS Forte supplements.

### Compliance

Vitamin and mineral deficiencies are common both before and after LRYGB surgery. Therefore additional supplements are advised after weight-loss surgery. However, life-long compliance of daily supplement intake is hard to achieve. To measure adequate intake of MVS, one can monitor the serum concentration of highly absorbable vitamins. Navarro et al^48^ demonstrated a serum folic acid concentration of five-fold compared with baseline, 4 hours after oral intake of standard MVS containing 1.6 mg folic acid. Therefore, folic acid can be used as a marker for compliance of MVS intake.

The capsules in both groups had a cherry flavor with powder, nevertheless, several patients complained about vitamin B aftertaste for a period of 30 minutes after ingestion. This had no influence for the compliance of the patients. After the end of the study, commercially available capsules have a bubblegum taste with microcapsulation to prevent this aftertaste.∗∗

### Limitations

Despite the double-blind, prospective, randomized character of the study, several limitations of the study should be considered. High prevalence of preoperative nutritional and mineral deficiencies, which are linked to morbid obesity, required additional treatment, and some patients received additional supplements after surgery outside of protocol. This will have obscured differences of the suppletion regimens to some extent. In Table 4, all patients receiving extra supplements have been excluded and, therefore, these data reflect the comparison of standard and optimized supplements most accurately. The demonstrated deficiencies develop gradually and progressively. Because of adequate reserves in the human body, many vitamin and nutrient deficiencies only occur after a few years. Although a significant difference in the first year has been shown in this study, we expect an even greater effect of optimized supplements compared with standard supplements in the long term. It is still uncertain whether customized multivitamin supplements can completely prevent postsurgical deficiencies. Continued surveillance is therefore needed to evaluate the effectiveness and safety on the long term, as patients will need life-long supplements after bariatric surgery.^49,50^ For example, excess vitamin B12 intake may be associated with prostate cancer.^50^

The compliance of patients was inquired during the follow-up interviews; however, the answers depended on the honesty of the patients and could only be compared with their folic acid levels. On the contrary, patients were blinded for the nature of their supplements and, thus, it is plausible that any noncompliant patients were equally distributed in both groups.

## CONCLUSION

In summary, prevention of mineral and vitamin deficiencies after LRYGB is hard to achieve with standard multivitamin supplements, especially when deficits before the operation are present. WLS Forte was introduced as a customized multivitamin supplement for post-RYGB intake, including 14,000% RDA (350 μg) vitamin B12, 500% RDA (70 mg) iron, and 300% RDA (600 μg) folic acid. Using WLS Forte after LRYGB surgery results in fewer deficiencies in iron, vitamin B12, and folic acid compared with sMVS. Despite the decreased risk of vitamin deficiencies with WLS Forte supplements after LRYGB, a strict follow-up regime remains necessary to prevent patients becoming vitamin deficient.
